# How patient, infection and cysticercus characteristics impact the evolution of *Taenia solium* larva in the human brain: A unique cyst-level analysis

**DOI:** 10.1017/S003118202400163X

**Published:** 2025-07

**Authors:** Hongbin Zhang, Meghana G. Shamsunder, Pryanka Bawa, Arturo Carpio, W. Allen Hauser, Karina Quinde-Herrera, Alex Jaramillo, Elizabeth A. Kelvin

**Affiliations:** 1 Department of Biostatistics, College of Public Health, University of Kentucky, Lexington, KY, USA; 2 CUNY Institute for Implementation Science in Population Health, City University of New York, New York, NY, USA; 3 Department of Epidemiology & Biostatistics, CUNY Graduate School of Public Health and Health Policy, City University of New York, New York, NY, USA; 4 School of Medicine, University of Cuenca, Cuenca, Ecuador; 5 G.H. Sergievsky Center, Vagelos College of Physicians and Surgeons, Columbia University, New York, NY, USA; 6 Department of Epidemiology, Mailman School of Public Health, Columbia University, New York, NY, USA; 7 Instituto de Diagnóstico por Imágenes, Cuenca, Ecuador; 8 Department of Occupational Health, Epidemiology & Prevention, Donald and Barbara Zucker School of Medicine at Hofstra University/Northwell Health, Hempstead, NY, USA

**Keywords:** albendazole, Ecuador, multistate modelling, neurocysticercosis, *Taenia solium*

## Abstract

Neurocysticercosis is a poorly understood infection of the central nervous system with *Taenia solium* larva, and the treatment often fails to kill all the parasitic larva. Most research on this infection has used patient-level data, looking at summaries of the encysted parasitic cysticercus burden. Cyst-level analysis is needed to identify factors that impact individual cyst trajectories and how that may vary based on characteristics of the patient, infection and cyst being followed. We disaggregated data on 221 cysts from 117 patients who participated in a trial evaluating the impact of albendazole treatment to identify factors that impact cyst evolution over time from the active to the degenerating and calcified phases, and eventual resolution. We found that having calcified cysts at baseline was associated with a faster rate of transition from the degenerative phase to calcified phase or resolution. Age and sex were not associated with cyst evolution in the main effect analysis, but after stratifying on treatment we found that the direction of some associations by patient age and sex was reversed for patients in the albendazole arm compared to those in the placebo arm. These findings suggest that differences in host immune response by sex and age as well as by past exposure, potentially indicated by having calcified cysts together with active cysts at baseline, are important to cyst evolution and may be modified by treatment. Future research is needed to assess if these differences suggest distinct treatment recommendations.

## Graphical abstract

**Figure:**
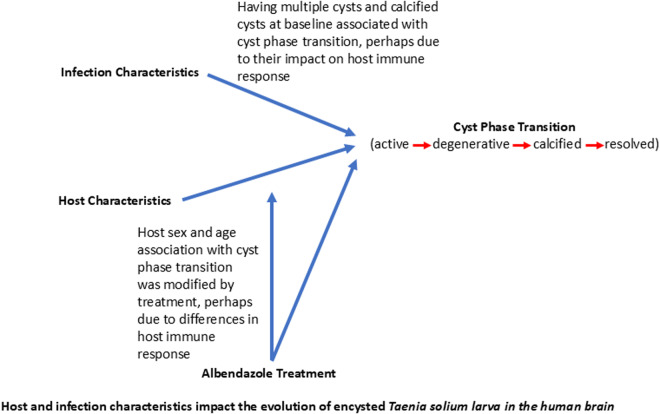

## Introduction

Neurocysticercosis (NCC) is an infection of the central nervous system with the larval stage of the pork tapeworm, *Taenia solium* (*T. solium*). Humans become infected with *T. solium* larva through the fecal oral route, usually by consuming food contaminated by *T. solium* eggs or gravid proglottids, which are shed by human carriers of the adult tapeworm. NCC can be completely asymptomatic or present with a number of neurological symptoms, the most common of which are seizures and headache (Hamamoto Filho et al., 2022). NCC is endemic in many low- and middle-income countries across Latin America, sub-Saharan Africa and Asia (Hamamoto Filho et al., 2022), and it is becoming more common in the United States (US) and Canada due to increased immigration and international travel (Gripper and Welburn, 2017). The World Health Organization (WHO) estimates that 2.56–8.30 million people are infected with NCC worldwide (WHO, 2019), and the Centers for Disease Control and Prevention (CDC) reports approximately 1000 NCC-related hospitalizations occur in the US each year (CDC., 2023). Despite this, research on NCC is limited and it remains a neglected tropical disease (WHO, 2019; CDC., 2023).

In the brain, *T. solium* larvae appear to go through 3 phases of evolution (Carpio et al., 1994). The active phase, in which the parasite larva is viable or alive, the degenerating phase, where the host immune system mounts an immune response against the parasitic larva that may lead to increased edema around the cysticercus (cyst) and, lastly, the deceased parasite can either resolve (disappear entirely) or become calcified (calcification) (Montgomery et al., 2019), possibly eliciting continued immune responses and additional symptoms (Gupta et al., 2002). One study found that calcified cysts sometimes resolve, but this is thought to be uncommon (Meneses Quiroz et al., 2015).

For patients with NCC cysts in the active phase, anthelminthic medication (albendazole or praziquantel), in conjunction with corticosteroids to reduce inflammation, is the recommended treatment regimen (White et al., 2018; WHO, 2021). Note these guidelines are informed by the trial on albendazole treatment from which the data used in this analysis came (Carpio et al., 2008). For patients with more than 2 cysts, dual treatment with albendazole and praziquantel is recommended (White et al., 2018; WHO, 2021). However, this regiment is not effective in all patients, with only about 31% of patients with active cysts having all parasites deceased 1 month following treatment. Therefore, the majority of patients with active cysts continue to have viable parasitic larva despite being treated. The impact of treatment is weaker on degenerating cysts, with only 27% of patients with degenerating cysts having them all resolved 1 month following treatment (Carpio et al., 2008). The impact of treatment also varies by cyst location and number (White et al., 2018). Studies that have looked at the impact of anthelmintic treatment on clinical symptoms have shown only small benefits (Garcia et al., 2004; Romo et al., 2015; Thapa et al., 2018), and the relationship appears to be complicated, with the benefit of treatment changing over time as NCC cysts resolve naturally among those who did not receive anthelmintic drugs (Carpio et al., 2019). Studies suggest that patient factors may also impact the ability of the host immune system to detect and target NCC cysts. Both sex and age differences in immune response have been described (Fleury et al., 2004; Kelvin et al., 2009).

Most studies on NCC have looked at cysts in aggregated form, focusing on changes in the total number of NCC cysts in the brain by phase (e.g. number of active cysts). However, research has found that individual cysts in the same person can evolve differently (Carpio et al., 1998). This suggests that, in addition to treatment and patient characteristics, factors related to the individual cyst, such as location, as well as the infection, such as number, phase or proximity of other NCC cysts, may influence cyst evolution. These factors can only be explored in a cyst-level analysis in which individual cysts within the same person are followed over time. Cyst-level analyses have generally only been conducted among patients with a single NCC cyst at baseline (Nandhagopal, 2011). However, in 1 study, data were disaggregated from the patient to the cyst-level for each cyst that was the only one in a specific brain location and then followed those cysts over time to assess where in the evolutionary process albendazole treatment works (Montgomery et al., 2019). What made the study unique is that it followed individual cysts in patients with multiple NCC cysts, although those other cysts had to be in other brain locations. The study found that albendazole impacts cyst evolution from the active to the degenerating phase as well as from the degenerating phase to resolution, but not to calcification, indicating that albendazole does not increase risk of cyst calcification. Furthermore, the impact of treatment varied by patient age and sex as well as cyst location and the presence of calcified cysts at baseline (Montgomery et al., 2019). This suggests that factors that could influence the host response to NCC may modify the impact of treatment, but that study did not describe the main effect of patient, infection or cyst characteristics. A better understanding of what impacts NCC cyst evolution for the total population and within each treated and placebo subpopulation is needed in order to inform the development of personalized therapies to improve outcomes for people with NCC (Toledo et al., 2018).

Therefore, the aims of this study were to assess the impact of characteristics of the host (age and sex), the NCC infection (one vs 2+ NCC cysts in the brain and presence of calcified cysts at baseline) and the cyst itself (location) on NCC cyst evolution over time. In addition, we assessed if this impact is modified by albendazole treatment by conducting a cyst-level analysis among NCC patients with diverse presentations in terms of the number, phase and location of NCC cysts and symptoms experienced.

## Materials and methods

### Data source and study design

Data for these analyses came from a randomized, double-blind, placebo-controlled clinical trial (RCT) on the impact of albendazole treatment vs placebo on NCC cyst resolution. The study was registered with ClinicalTrials.gov (#NCT00283699) (Hauser, 2006) and has been previously described (Carpio et al., 2008). Briefly, patients were recruited in February 2001–February 2003 from 6 hospitals in Ecuador and were eligible for the study if they presented with new onset symptoms consistent with NCC (for a description of symptoms, see Thapa et al. (2018)) and were diagnosed with NCC based on computed tomography (CT) or magnetic resonance imaging (MRI) with evidence of active and/or degenerating NCC cysts. The diagnostic criteria used for this study have been previously described (Carpio et al., 2008). Patients were ineligible if they only had calcified cysts, were pregnant, had another progressive or life-threatening disorder, had received treatment for NCC within the past year or had received systemic treatment with steroids in the past month. Partway through the study, patients with ventricular shunt were excluded for safety reasons. Patients were randomized to receive albendazole (400 mg) or placebo, both given orally every 12 h for 8 days. All patients received prednisone (75 mg), daily for 8 days, then tapered off over 2 weeks and treatment for the symptoms they were experiencing (e.g. antiseizure medication for those with seizures). Patients had CT (thickness of approximately 3–5 mm for infratentorial cuts and 5 × 8 mm for supratentorial cuts) or MRI scans at baseline, 1, 6, 12 and 24 months of follow-up. For each scan, a radiologist documented the number of cysts in each phase (active, degenerative or calcified) by brain location and the count for the brain as a whole.

All adult participants provided signed informed consent. Guardians/parents of paediatric patients provided signed informed consent and the child provided oral assent. Ethics review and approval was granted by the Institutional Review Board (IRB) of Columbia University, the Office of Human Research Protection of the National Institutes of Health, as well as the ethics committees at each participating hospital.

### Data disaggregation for cyst-level analysis

In order to follow the evolution of individual NCC cysts over time, we disaggregated data from the patient- to the cyst-level for all cysts that were the only ones in a specific brain location at baseline (e.g. in the right temporal lobe) in order to be able to follow the cysts through the 24 month follow-up without accidentally mixing them up with other cysts in the same location. Any cysts that appeared to reverse transition (*n* = 14), meaning movement from one evolutionary phase backward to an earlier phase (i.e. from the degenerating phase to the active phase) were excluded from analysis under the assumption that this was an error in the data. This process yielded a dataset of 221 total cysts in 117 different patients (Montgomery et al., 2019; Zhang et al., 2020).

### Measures

The outcome of interest was cyst evolution from one phase to another (e.g. from active to degenerating or active to calcification). We assumed that cysts could skip phases due to the time gap between imaging.

We looked at 3 types of predictors of cyst evolution: (1) patient characteristics, (2) infection characteristics and (3) cyst characteristics. For patient characteristics we looked at age, dichotomized at the median of 40 years at study enrollment and sex (female vs male). For infection characteristics, we looked at an indicator for having more than 1 NCC cysts in the brain at baseline (vs only the one we were following). For patients with multiple NCC cysts, the number of cysts ranged from 2 to 50+ (the radiologist stopped counting at 50 cysts) in any phase, with many participants having cysts in multiple phases at baseline. We also looked at an indicator for having 1 or more calcified NCC cysts at baseline, which may predict the probability that other NCC cysts calcify rather than resolve completely. For cyst characteristics, we looked at whether the individual cyst being followed was in an extraparenchymal region of the brain (vs parenchymal region). We also included treatment arm (albendazole vs placebo), which is known to impact NCC cyst evolution (i.e. killing the parasite so it moves from the active to the degenerative phase), and which may interact with patient, infection and cyst characteristics in impacting cyst evolution.

### Statistical analysis

We described the study sample in terms of patient, infection and cyst characteristics overall and the correlation between variables to describe the within group distribution. We also summarized the evolution of the NCC cysts over time. Then we ran a series of multistate models (MSMs) of increasing complexity to assess the associations among patient, infection and cyst characteristics as well as albendazole treatment with NCC cyst evolution over time. MSMs are an extension of the survival analysis methods where both the risk of overall survival and the instantaneous risk for intermediate transitions are of interest. A comprehensive overview of MSMs (Anderson and Keiding, 2002; Putter et al., 2007) as well as how we use them have been previously described (Zhang et al., 2020), but briefly, we used a 4-state multistate Markov model with 3 transient states (active, degenerative and calcification) and 1 absorbing state (cyst resolution) to model the hazards of changing to subsequent phases over the study period. Due to the fixed imaging schedule (baseline, months 1, 6, 12 and 24), the exact time of a phase change is unknown. For example, if a patient exhibited a cyst change from degeneration to resolution between months 1 and 6, the exact time of that change during the 5-month period is unknown. In addition, we observed certain ‘direct’ evolutions, for example, cysts that were observed to ‘progress’ from the active phase to resolution directly, while intermittent states (i.e. the degenerative phase) are assumed to have occurred in the interim although they were not observed. This problem is addressed with an interval-censoring schema in MSMs. In addition, since in some cases we were following multiple NCC cysts in the same patient and those evolutions were likely correlated, we used Bootstrapping to estimate 95% confidence intervals (CI) taking the correlation into account. All analyses were conducted in R 4.2.1 (R Foundation of Statistical Computing, Vienna, Austria) using the MSM (Jackson, 2011) package.

First, we ran separate crude MSMs for each variable of interest. We then ran an MSM including only patient characteristics and treatment and another MSM including infection and cyst characteristics and treatment. We then ran a third model including all variables. Such incremental modelling allows us to examine the multiple confounding issues in different scenarios. We then re-run the analysis to fit those incremental models described above by treatment strata (i.e. 1 strata among those who received albendazole and 1 strata among those who received placebo) to see how the impact of patient, infection and cyst characteristics are modified by treatment.

## Results

### Description of the sample

Of the 117 participants included in our analysis, 62 (53%) were male, 107 (48%) were aged 40 years (the median age) or older and 62 (53%) received albendazole treatment. At baseline, the majority (*n* = 86, 74%) had more than 1 NCC cyst in their brain and 45 (38%) had 1 or more calcified cysts. The majority of cysts followed in this analysis were situated within parenchymal regions of the brain, accounting for 105 cases (90%) (Table 1).

**Table 1.** Description of patient, infection and cyst characteristics

Characteristics of the sample	Patient *n* (%)
Total	117 (100)
Sex	
Male	62 (53)
Female	55 (47)
Age in years	
≥40	107 (48)
< 40	115 (52)
Number of NCC cysts in the brain at baseline	
2+	86 (74)
1	31 (26)
Had calcified NCC cysts at baseline?	
Yes	45 (38)
No	72 (62)
Cyst being followed was in parenchymal region of the brain	
Yes	105 (90)
No^a^	12(10)

a Extraparenchymal cysts included those that were in intraventricular, cisternal and subarachnoid locations.

Table 2 shows the correlation among the variables included in these analyses. For example, among female participants, 44% were ≥40 years old, while among male participants, 58% were ≥40 years old. In addition, 46% of participants <40 years old were male, while 60% of those ≥40 years old were male (Table 2).

**Table 2.** Within-group distribution of study variables

	Patient characteristics				Infection characteristics				Cyst characteristic	
	Sex		Age (years)		Had 2+NCC cysts at baseline		Had calcified NCC cysts at baseline		Cyst being followed was in parenchymal location	
Covariate	Female	Male	<40	≥40	No	Yes	No	Yes	No	Yes
Male sex			26(46%)	36(60%)	15(48%)	47(55%)	32(44%)	30(67%)	5(42%)	57(54%)
≥40 years of age	24(44%)	36(58%)			11(35%)	49(57%)	37(51%)	23(51%)	6(50%)	54(51%)
2+ NCC cysts in the brain at baseline	39(71%)	47(76%)	37(65%)	49(82%)			41(57%)	45(100%)	8(67%)	78(74%)
Calcified cysts present at baseline	15(27%)	30(48%)	22(39%)	23(38%)	0(0%)	45(52%)			1(8%)	44(42%)
Cyst being followed is located in parenchymal locations	48(87%)	57(92%)	51(89%)	54(90%)	27(87%)	78(91%)	61(85%)	44(98%)		
Albendazole treatment received	28(51%)	34(55%)	33(58%)	29(48%)	15(48%)	47(55%)	37(51%)	25(56%)	8(67%)	54(51%)

Over the 24-month follow-up, 203 cysts transited to subsequent evolutionary phases (43.9% of all observations). This includes 95 transitions from the active phase to subsequent phases (49.5% of active cyst observations transitioned to the degenerative or calcified phase or resolved), 73 transitions of degenerative cysts to subsequent phases (41.5% of degenerative cysts transitioned to calcified phase or resolved during follow-up) and 35 (31.8%) of calcified cysts resolved (Table 3).

**Table 3.** Description of the number of cyst transitions across evolutionary phases observed over 24-month follow-up

	To state					Overall change	
		**Active**	**Degenerative**	**Calcified**	**Resolved**	**No phase change, *n* (%)**	**Transitioned to new phase, *n* (%)**
**From state**	Active	97	19	2	74	97 (50.5)	95 (49.5)
	Degenerative	0	89	7	66	89 (54.9)	73 (45.1)
	Calcified	0	0	75	35	75 (68.2)	35 (31.8)
	All cysts	97	108	84	175	261 (56.3)	203 (43.9)

### *MSM results (refer to* *Appendix Table 1* *for a summary of the connection between models, hypotheses tested and main findings)*

In the crude MSMs (Table 4), participant characteristics (sex and age) were not significantly associated with cyst transitioning, but characteristics of the infection and the cyst being followed were. Cysts located in participants with multiple NCC cysts in their brain at baseline had a significantly higher rate of transitioning from the active to the degenerative phase (hazard ratio [HR] = 2.99, 95% CI: 1.28–9.07) and cysts in participants who had calcified cysts in their brain at baseline had a higher rate of transitioning from the degenerative phase to resolution (the cyst disappeared) (HR = 2.35, 95% CI: 1.13–6.67). In addition, cysts located in the parenchymal region had 1.76 times higher rate of transitioning from the degenerative phase to calcification (95% CI: 1.08–6.50) but a lower rate of transitioning from the calcified phase to resolution (HR = 0.14, 95% CI: 0.01–0.96). As previously reported (Montgomery et al., 2019), cysts in patients treated with albendazole had significantly higher rates of transition from the active to the degenerative phase and from the degenerative phase to resolution (HR = 2.90, 95% CI: 1.43–6.79; HR = 1.92, 95% CI: 1.03–4.42, respectively).

**Table 4.** Crude MSM results for the association of patient, infection and cyst characteristics and albendazole treatment with NCC cyst transitions across the 4 phases of evolution over 24 months

	Hazard ratio (95% confidence interval) for cyst evolution from one phase to another			
Variable	Active → degenerative	Degenerative → calcified	Degenerative → resolution	Calcified → resolution
Participant characteristics				
Male sex (ref = female)	0.99 (0.40, 2.13)	1.08 (0.19, 3.79)	1.21 (0.70, 2.51)	0.56 (0.22, 1.16)
≥40 years of age (ref = <40)	1.03 (0.44, 2.15)	0.39 (0.02, 2.21)	0.61 (0.31, 1.16)	1.04 (0.52, 2.59)
2+ NCC cysts in brain at baseline (ref = 1 cyst)	2.99 (1.28, 9.07)^a^	0.70 (0.18, 9.27)	1.84 (0.87, 4.11)	1.48 (0.31, 26.59)
Had calcified NCC cysts at baseline (ref = no)	1.53 (0.69, 3.64)	4.93 (0.86, 84.21)	2.35 (1.13, 6.67)^a^	0.90 (0.10, 22.47)
Cyst characteristic				
Cyst located in parenchymal region (ref = extraparenchymal location)	0.62 (0.15, 1.83)	1.76 (1.08, 6.50)^a^	0.37 (0.05, 1.07)	0.14 (0.01, 0.96)^a^
Albendazole treatment (ref = placebo)	2.90 (1.43, 6.79)^a^	0.99 (0.05, 3.95)	1.92 (1.03, 4.42)^a^	(0.48, 2.15)

a *P* < 0.05.

In the multivariable MSM with patient characteristics and treatment, only albendazole treatment was significantly associated with cyst transitioning (active to degenerative HR = 3.75, 95% CI: 1.32–13.21). In the model with only infection and cyst characteristics and treatment, having calcified cysts in the brain at baseline was significantly associated with evolution from the degenerative phase to resolution (HR = 3.33, 95% CI: 1.35–316.75), and cysts located in parenchymal regions had lower rate of transitioning from calcified to resolution (HR = 0.14, 95% CI: 0.01–0.95). Albendazole treatment remained positively associated with transition from the active to the degenerative phase and degenerative to resolution (HR = 3.31, 95% CI: 1.77–14.15; HR = 2.92, 95% CI: 1.10–178.67, respectively), but the estimates for the degenerative to resolution transition had a large confidence interval due to high variability due to the model complexity and relative small sample. In the full model including patient, infection and cyst characteristics plus treatment, having calcified cysts at baseline was associated with higher rate of transition from the degenerative phase to cyst resolution (HR = 4.33, 95% CI: 1.24–367.21), and albendazole treatment remained associated with transitioning from the active to the degenerative phase (HR = 5.13, 95% CI: 1.45–19.52), but the transition from the degenerative phase to cyst resolution was no longer significant (Table 5).

**Table 5.** Multivariable MSM results for the association of patient, infection and cyst characteristics and albendazole treatment with NCC cyst transitions across the 4 phases of evolution over 24 months

	Hazard ratio (95% confidence interval) for cyst evolution from one phase to another			
Variable	Active → degenerative	Degenerative → calcified	Degenerative → resolution	Calcified → resolution
**Model with only patient characteristics and treatment**				
Male sex (ref = female)	1.46 (0.41, 5.22)	1.50 (0.05, 16.69)	1.66 (0.67, 8.84)	0.37 (0.07, 1.28)
≥40 years of age (ref = <40)	0.90 (0.29, 2.26)	0.22 (0.01, 3.03)	0.71 (0.26, 3.57)	1.46 (0.44, 5.38)
Albendazole treatment (ref = placebo)	3.75 (1.32, 13.21)^a^	0.49 (0.01, 4.04)	1.97 (0.96, 12.94)	1.39 (0.45, 4.04)
**Model with only infection and cysts characteristics and treatment**				
2+ NCC cysts in brain at baseline (ref = 1 cyst)	2.92 (0.83, 11.44)	0.03 (0.01, 3.10)	1.28 (0.33, 3.51)	5.05 (0.05, 374.31)
Had calcified NCC cysts at baseline (ref = no)	1.39 (0.45, 4.58)	135.60 (0.13, 848.29)	3.33 (1.35, 316.75)^a^^,^^b^	0.51 (0.01, 23.74)
Cyst located in parenchymal region (ref = extraparenchymal location)	0.48 (0.12, 1.72)	1.73 (0.01, 32.32)	0.27 (0.01, 12.67)	0.14 (0.01, 0.95)^a^
Albendazole treatment (ref = placebo)	3.31 (1.77, 14.15)^a^	1.76 (0.01, 17.10)	2.92 (1.10, 178.67)^a^^,^^b^	0.93 (0.38, 2.09)
**Full model with patient, infection and cyst characteristics and treatment**				
Male sex (ref = female)	1.84 (0.42, 8.43)	1.55 (0.07, 16.47)	1.70 (0.63, 11.86)	0.50 (0.04, 2.28)
≥40 years of age (ref = <40)	0.78 (0.24, 1.94)	0.11 (0.01, 8.32)	0.48 (0.06, 5.72)	1.02 (0.22, 4.63)
2+ NCC cysts in brain at baseline (ref = 1 cyst)	5.16 (0.82, 24.49)	0.02 (0.01, 4.28)	1.22 (0.22, 5.80)	4.84 (0.02, 1967.03)
Had calcified NCC cysts at baseline (ref = no)	1.07 (0.35, 4.36)	260.18 (0.12, 3218.84)	4.33 (1.24, 367.21)^a^^,^^b^	0.58 (0.01, 70.50)
Cyst located in parenchymal region (ref = extraparenchymal location)	0.46 (0.11, 1.72)	2.36 (0.01, 9.61)	0.24 (0.01, 32.68)	0.29 (0.01, 5.23)
Albendazole treatment (ref = placebo)	5.13 (1.45, 19.52)^a^	0.67 (0.01, 7.84)	2.17 (0.84, 321.73)	1.05 (0.28, 4.59)

a *P* < 0.05.

b Large confidence interval due to high standard error due to substantial variability caused by small samples in the data for the comparison.

### Stratified MSM results

When we reran the crude models stratified on treatment, among patients who received albendazole, there was a non-significant higher rate of cyst transitioning from degenerative to calcified and degenerative to resolution for male patients compared to female patients, while the direction of the association between sex and cyst transitioning reversed among those in the placebo group, with a significantly lower rate of transitioning from degenerating to resolution among men (HR = 0.29, 95% CI: 0.12–0.60). The direction of the association by age also varied by strata. Among those who received albendazole, patients in the older age group had higher rate of transitioning across all phases except for active to degenerative, for which the transition rate was lower, while among those in the placebo group, the results were the opposite with older patients having a lower rate of transitioning across all phases except active to degenerative, for which they had a higher rate of transition. Characteristics of the infection (multiple cysts and presence of calcified cysts at baseline) and location of the cyst being followed also showed patterns that suggest effect modification by treatment. For example, among those in the albendazole group, having multiple cysts at baseline was associated with significantly lower transition rate for the calcified to resolution transition (HR = 0.46, 95% CI: 0.21–0.94) but a non-significant higher rate for those in the placebo group (HR = 23.61, 95% CI: 0.52–84.56). Among those in the albendazole group, having calcified cysts at baseline remained significantly positively associated with transition from degenerating to resolution (HR = 7.84, 95% CI: 2.81–858.12) and slower transition from calcified to resolution (HR = 0.13, 95% CI: 0.01–0.45), while among those in the placebo group these transitions were weaker (degenerative to resolve HR = 1.23, 95% CI: 0.47–4.41) or in the opposite direction (calcified to resolution HR = 21.79, 95% CI: 0.64–69.24) but non-significant. While we saw the location impacted multiple transition before stratification (Table 4), in the stratified analysis the significant association was among those in the placebo group where cysts in parenchymal regions had a higher rate of transitioning from degenerating to calcified (HR = 2.82, 95% CI: 1.04–9.3), but there was no strong pattern suggesting effect modification by treatment (Table 6).

**Table 6.** Crude MSM results for the association of patient, infection and cyst characteristics and albendazole treatment with NCC cyst transitions across the 4 phases of evolution over 24 months stratified on treatment

	Albendazole Hazard ratio (95% confidence interval) for cyst evolution from one phase to another				Placebo Hazard ratio (95% confidence interval) for cyst evolution from one phase to another			
Variable	Active → degenerative	Degenerative → calcified	Degenerative → resolution	Calcified → resolution	Active → degenerative	Degenerative → calcified	Degenerative → resolution	Calcified → resolution
Male sex (ref = female)	1.26 (0.18, 3.92)	2.47 (0.04, 142.56)	2.27 (0.58, 8.48)	2.26 (0.32, 147.29)	1.80 (0.83, 5.53)	0.11 (0.01, 1.60)	0.29 (0.12, 0.60)^a^	1.08 (0.01, 6.88)
≥40 years of age (ref = < 40)	0.72 (0.18, 2.41)	1.20 (0.05, 96.24)	1.87 (0.59, 7.65)	1.55 (0.57, 7.72)	1.53 (0.54, 4.44)	0.12 (0.01, 2.11)	0.31 (0.10, 0.73)	0.63 (0.13, 2.21)
2+ NCC cysts in brain at baseline (ref = 1 cyst)	4.25 (0.62, 21.17)	1.03 (0.03, 27.26)	2.49 (0.28, 7.21)	0.46 (0.21, 0.94)^a^	3.02 (0.58, 151.49)	0.49 (0.01, 15.16)	1.46 (0.48, 3.99)	23.61 (0.52, 84.56)
Had calcified NCC cysts at baseline (ref = no)	1.74 (0.34, 6.37)	20.57 (0.59, 2649.46)	7.84 (2.81, 858.12)^a^^,^^b^	0.13 (0.01, 0.45)^a^	2.11 (0.88, 4.40)	3.64 (0.18, 158.05)	1.23 (0.47, 4.41)	21.79 (0.64, 69.24)
Cyst located in parenchymal region (ref = extraparenchymal location)	0.35 (0.03, 6.83)	1.21 (0.92, 2.42)	0.15 (0.01, 0.46)	0.17 (0.01, 1.00)	0.84 (0.33, 1.86)	2.82 (1.04, 9.3)^a^	1.03 (0.02, 58.71)	0.88 (0.64, 1.01)

a *P* < 0.05.

b Large confidence interval due to high standard error due to substantial variability caused by small samples in the data for the comparison.

In the full stratified multivariable MSMs, men had higher rates of transitions across all phases than women when treated with albendazole (active to degenerative HR = 5.78, 95% CI: 0.58–80.80; degenerative to calcified HR = 10.79, 95% CI: 0.01–2554.93); degenerative-resolved HR = 2.01, 95% CI: 0.01–1057.80; calcified to resolved HR = 1.06, 95% CI: 0.01–226.94) but lower rates when treated with placebo (HR = 0.29, 95% CI: 0.01–2.06; HR = 0.75, 95% CI: 0.01–86.25; HR = 1.09, 95% CI: 0.10–3.75; HR = 0.45, 95% CI: 0.01–13.20, respectively), but none of the associations were statistically significant. The pattern with age in the crude model was no longer apparent, but having calcified cysts at baseline was associated with a reduced rate of transition from active to degenerative phase among those treated with albendazole (HR = 0.43, 95% CI: 0.07–3.82) but a higher rate among those treated with placebo (HR = 2.11, 95% CI: 0.22–41.56), but these associations were not statistically significant (Table 7).

**Table 7.** Multivariable MSM results for the association of patient, infection and cyst characteristics with NCC cyst transitions across the 4 phases of evolution over 24 months stratified on treatment

	Albendazole Hazard ratio (95% confidence interval) for cyst evolution from one phase to another				Placebo Hazard ratio (95% confidence interval) for cyst evolution from one phase to another			
Variable	Active → degenerative	Degenerative → calcified	Degenerative → resolution	Calcified → resolution	Active → degenerative	Degenerative → calcified	Degenerative → resolution	Calcified → resolution
**Model with patient characteristics**								
Male sex (ref = female)	2.32 (0.51, 21.51)	5.38 (0.04, 534.21)	2.45 (0.78, 16.42)	0.39 (0.01, 1.46)	0.56 (0.06, 2.15)	0.67 (0.02, 30.88)	0.99 (0.32, 4.78)	0.40 (0.03, 9.41)
≥40 years of age (ref = <40)	0.63 (0.08, 2.34)	0.81 (0.03, 82.55)	1.52 (0.29, 6.70)	1.84 (0.52, 13.04)	1.63 (0.35,4.85)	0.14 (0.01, 4.26)	0.31 (0.08, 1.34)	1.08 (0.05, 13.41)
**Model with infection and cysts characteristics**								
2+ NCC cysts in brain at baseline (ref = 1 cyst)	4.52 (0.02, 16.19)	0.07 (0.01, 4.42)	1.28 (0.19, 4.05)	1.38 (0.02, 40.11)	4.46 (0.48, 22.28)	0.06 (0.01, 5.69)	1.20 (0.22, 9.34)	0.98 (0.03, 44.95)
Had calcified NCC cysts at baseline (ref = no)	1.00 (0.28, 5.81)	115.33 (1.58, 17961.0)^a^^,^^b^	8.61 (3.06, 2375.39)^a^^,^^b^	0.12 (0.01, 17.88)	1.16 (0.24, 4.29)	159.25 (2.51, 30631.75)^a^^,^^b^	9.37 (2.30, 1310.22)^a^^,^^b^	0.12 (0.01, 17.29)
Cyst located in parenchymal region (ref = extraparenchymal location)	0.49 (0.01, 9.40)	1.53 (0.01, 6.58)	0.13 (0.01, 0.89)	0.38 (0.02, 1.78)	0.58 (0.01, 2.58)	1.55 (0.01, 8.78)	0.13 (0.01, 0.69)	0.38 (0.01, 1.80)
**Model with patient, infection and cysts characteristics (full model)**								
Male sex (ref = female)	5.78 (0.58, 80.80)	10.79 (0.01, 2554.93)	2.01 (0.01, 1057.80)	1.06 (0.01, 226.94)	0.29 (0.01, 2.06)	0.75 (0.01, 86.25)	1.09 (0.10, 3.75)	0.45 (0.01, 13.20)
≥40 years of age (ref = <40)	0.36 (0.02, 1.87)	0.44 (0.01, 898.75)	0.71 (0.01, 99.18)	1.39 (0.35, 13.29)	1.15 (0.09,5.95)	0.05 (0.01, 4.62)	0.27 (0.05, 2.01)	0.48 (0.01, 31.49)
2+ NCC cysts in brain at baseline (ref = 1 cyst)	13.66 (0.01, 451.42)	0.38 (0.01,20.51)	1.69 (0.01, 558.07)	0.89 (0.01, 2986.57)	3.27 (0.38, 1046.17)	0.02 (0.01, 3.86)	1.43 (0.38, 8.84)	13.19 (0.32, 989.93)
Had calcified NCC cysts at baseline (ref = no)	0.43 (0.07, 3.82)	45.18 (0.02, 71369.65.0)	60.13 (0.61, 200155.89)	0.07 (0.01, 59.27)	2.11 (0.22, 41.56)	194.41 (1.16, 3438.32)^a^^,^^b^	1.42 (0.21, 6.34)	22.64 (0.28, 725.77)
Cyst located in parenchymal region (ref = extraparenchymal location)	0.15 (0.01, 6.97)	1.07 (0.01, 144.91)	0.09 (0.01, 12.23)	0.31 (0.01, 178.80)	0.33 (0.01, 3.70)	2.06 (0.10, 54.82)	1.16 (0.01, 172.75)	0.89 (0.08, 0.99)^a^

a *P* < 0.05.

b Large confidence interval due to high standard error due to substantial variability caused by small samples in the data for the comparison.

Because calcified cysts and cyst disappearance may both be considered resolution of the infection, and calcified cysts may be more difficult to detect due to their smaller size making misclassification of resolution more common, we conducted a sensitivity analysis in which we combined cyst calcification and disappearance into 1 category and reran the stratified models. In these models, we again see a higher rate of transition among males (vs female) across the phases among those in the albendazole group and a lower rate among those in the placebo group. We also see age differences similar to those in the crude model in Table 6, with older patients treated with albendazole having a higher transition from active to degenerative and lower transition rate from degenerative to calcified or resolved (HR = 0.58, 95% CI: 0.08–2.29 & HR = 1.46, 95% CI: 0.36–6.62) but the opposite direction of association among those in the placebo group (HR = 1.64, 95% CI: 0.38–5.08 & HR = 0.29, 95% CI: 0.11–0.72). We also still see the negative association of having calcified cysts at baseline with transition from active to degenerative among those in the albendazole group and the positive association for those in the placebo group, similar to the full model in Table 7 (Table 8).

**Table 8.** Sensitivity analysis combining cyst calcification and resolution into 1 outcome for the multivariable MSM for the association of patient, infection and cyst characteristics with NCC cyst transitions across the 3 phases of evolution over 24 months stratified on treatment

	Albendazole Hazard ratio (95% confidence interval) for cyst evolution from one phase to another		Placebo Hazard ratio (95% confidence interval) for cyst evolution from one phase to another	
Variable	Active → degenerative	Degenerative → calcified or resolved	Active → degenerative	Degenerative → calcified or resolved
**Model with patient characteristics**				
Male sex (ref = female)	2.23 (0.48, 21.69)	2.55 (0.83, 14.08)	0.59 (0.05, 2.07)	0.96 (0.37, 2.03)
≥40 years of age (ref = <40)	0.58 (0.08, 2.29)	1.46 (0.36, 6.62)	1.64 (0.38, 5.08)	0.29 (0.11, 0.72)^a^
**Model with infection and cysts characteristics**				
2+ NCC cysts in brain at baseline (ref = 1 cyst)	3.69 (0.01, 16.77)	1.03 (0.17, 2.88)	3.75 (0.03, 27.55)	1.05 (0.13, 2.67)
Had calcified NCC cysts at baseline (ref = no)	0.86 (0.33, 4.95)	9.98 (4.22, 1273.62)^a^^,^^b^	1.27 (0.16, 5.97)	10.43 (3.58, 1635.82)^a^^,^^b^
Cyst located in parenchymal region (ref = extraparenchymal location)	0.57 (0.01, 7.98)	0.13 (0.01, 0.54)^a^	0.52 (0.01, 6.04)	0.14 (0.01, 0.44)^a^
**Model with patient, infection and cysts characteristics (full model)**				
Male sex (ref = female)	5.89 (0.75, 74.55)	2.30 (0.74, 14.09)	0.29 (0.01, 1.82)	1.09 (0.34, 2.92)
≥40 years of age (ref = <40)	0.35 (0.02, 1.81)	0.80 (0.20, 8.88)	1.16 (0.08, 6.18)	0.25 (0.06, 0.76)^a^
2+ NCC cysts in brain at baseline (ref = 1 cyst)	13.86 (0.09, 395.78)	0.97 (0.17, 3.48)	3.06 (0.31, 764.77)	1.11 (0.35, 5.76)
Had calcified NCC cysts at baseline (ref = no)	0.41 (0.05, 3.26)	10.55 (1.66, 2373.79)^a^^,^^b^	2.19 (0.17, 40.34)	1.77 (0.47, 5.61)
Cyst located in parenchymal region (ref = extraparenchymal location)	0.15 (0.01, 4.11)	0.11 (0.01, 0.69)^a^	0.33 (0.01, 3.98)	1.09 (0.03, 50.50)

a *P* < 0.05.

b Large confidence interval due to high standard error due to substantial variability caused by small samples in the data for the comparison.

## Discussion

NCC research has historically looked at information about cyst evolution aggregated at the level of the patient, and cyst-level analyses in the past had only been conducted among people with a single cyst at baseline (Nandhagopal, 2011). However, research has found that individual cysts in the same person can evolve differently (Carpio et al., 1998), suggesting that cyst-level analysis may be important to our understanding of this infection. Our analysis followed individual cysts in people who varied in terms of their characteristics (sex and age) and those of their infection (having a single or multiple NCC cysts and presence of calcified cysts at baseline) and the location of the cyst being followed. We found that characteristics of the infection were associated with rate of cyst transitioning across the phases. In the fully adjusted model, having calcified NCC cysts at baseline was associated with 4.33 times increased rate of transitioning from the degenerative phase to cyst resolution. Having calcified cysts together with active cysts at baseline might be an indicator of previous infection. It seems possible that previous exposure or infection might prime the host immune system to recognize NCC cysts sooner than occurs with a first infection. It is important to expand the sample of cysts to include those in brain locations with multiple NCC cysts to see how number of NCC cysts and presence of calcified cysts at baseline impact cyst evolution when these other cysts are in a proximate location vs farther away in the brain.

We did not find that host characteristics (age and sex) impacted NCC cyst trajectories in the main effect analysis. This was surprising as previous research using data on NCC cysts aggregated to the individual participant found that women are more likely to mount an immune response to NCC than men (Fleury et al., 2004; Kelvin et al., 2009). The association with age in previous studies suggests that immune response is stronger in younger people at baseline (Fleury et al., 2004; Kelvin et al., 2009) but stronger among older people during later follow-up (Kelvin et al., 2009). However, differences in the treatment in our sample, with some receiving albendazole and others placebo, might explain our inability to find sex and age differences over time if these differences are modified by treatment. Therefore, we stratified the models on treatment arm. In the stratified models, there was a pattern of association suggestive of effect modification by sex. In the fully adjusted model, men had higher rates of transitions across all phases than women when treated with albendazole but lower rates when treated with placebo. This seems consistent with previous research suggesting that women mount a stronger immune system than men (Fleury et al., 2004; Kelvin et al., 2009), such that the additional benefit from albendazole treatment is lower among women whose immune systems have already targeted the parasites prior to treatment. This pattern remained in the sensitivity analysis in which we combined calcified and resolved cysts into 1 outcome.

Age also demonstrated patterns consistent with effect modification in the stratified, although the pattern was less robust. In the crude model, older patients (>40 years) had a lower rate of transition from active to degenerative but higher rate of transition across all other evolutions compared to younger patients when treated with albendazole, but the associations were in the opposite direction among those treated with placebo. This pattern was less clear in the adjusted models and sensitivity analysis, but the difference in direction of association for transition from active to degenerative remained. A previous study found a stronger immune response at baseline among younger patients, (Fleury et al., 2004; Kelvin et al., 2009) and among older patients over follow-up (Kelvin et al., 2009). Given the patterns by sex, age and presence of calcified cysts at baseline, which may be proxies for differences in immune response, future research should consider exploring complex interaction among these factors and treatment to identify the subgroups of patients that benefit from treatment and those that do not to inform care decisions, but that would require a much larger sample size.

The impact of albendazole treatment on rate of transition from the active to degenerative phase and from the degenerative phase to resolution found in this cyst-level study is consistent with our previous analysis (Montgomery et al., 2019) and with previous studies looking at aggregated data on cysts. A number of trials (Del Brutto et al., 2006; Baird et al., 2013), including 2 considered high-quality (White et al., 2018) that looked at data on NCC cysts aggregated to the patient-level, found that albendazole was associated with higher likelihood of resolution of active (Garcia et al., 2004; Del Brutto et al., 2006; Carpio et al., 2008; Baird et al., 2013) and degenerative cysts compared to placebo (Garcia et al., 2004; Del Brutto et al., 2006).

This study has a number of limitations that should be considered in interpreting its results. First, participants were diagnosed with NCC, and therefore eligible for participation in this study, because they experienced NCC-related symptoms. People with NCC may be asymptomatic for a long time and some may never experience symptoms (Hamamoto Filho et al., 2022). Therefore, our analysis looks at cyst evolution starting at the time when symptoms were experienced rather than when participants were infected. In addition, all participants received prednisone and symptomatic treatment, which may have interacted with albendazole and a comparison of albendazole only vs placebo only may have led to different results. This treatment regimen is consistent with current recommendations (White et al., 2018) as corticosteroids reduce inflammatory complications in the brain, but there is some suggestion that use of corticosteroids may reduce the efficacy of anthelminthic treatment due to its immunosuppressive properties (Hamamoto Filho et al., 2021). The scan data included both CT and MRI scans. Calcifications are more detectable on CT scans while active and degenerative cysts are more easily detected on MRIs (Lerner et al., 2012), making misclassification of calcification-related evolutions potentially more likely in MRI readings and misclassification of other phases more likely in CT readings. It is also possible that cysts apparent on a previous image shrank over time and were no longer visible because of the thickness of the cuts of the CT scan, which may explain the unexpectedly large number of calcified cysts that disappeared. Resolution of calcified cysts has been previously reported, but over a longer time period (8–9 years) and is thought to be uncommon (Meneses Quiroz et al., 2015). Of course for the calcified cysts present at baseline in our study, we do not know how long they have been calcified. Furthermore, when we compared the cyst data generated for this study independently by 2 different radiologists, we found kappas ranging from 0.4 to 0.7 (Carpio et al., 2008), which is fair–good but suggests some misclassification is present. Inter-rater agreement of radiologist readings are known to be in the fair–moderate range for numerous diseases (Spivak and Pirouzmand, 2005; Wardlaw and Mielke, 2005; De Schryver et al., 2006), and this is also the case for NCC. For these analyses, we used the reading of one of the radiologists, which likely included some misclassification. The sample in this analysis was limited to cysts that were the only cyst in a specific brain location (e.g. in the right frontal lobe or left parietal lobe). We detected a crude association between the number of cysts in the brain overall and transition of the individual cysts we followed, but it is possible that the location of those other cysts (distance from the cyst being followed or located in the same brain region) is important, but we were unable to assess this with the given data. Third, our categorization of the variables was fairly blunt (e.g. dichotomizing age and number of cysts) and modelling these variables as numeric or polytomous variables might have different results. Because of our small sample size, we did not have sufficient statistical power to explore other ways of modelling variables. Also related to the sample size, we were likely underpowered to detect some associations, leading to type 2 errors. This was especially a challenge when stratifying the models on treatment and prohibited the exploration of complex interactive relationships among more than 2 variables (e.g. sex × age × treatment). In addition, follow-up scans were missing for some participants, which also contributed to reduced power and may have biased results if missing data were related to cyst transitions. Finally, the patients in this study were recruited from hospitals in Ecuador so these results may not be generalizable to NCC patients from other countries.

Despite these limitations, this is one of the first studies looking at the progression of NCC infection by following individual cysts rather than summaries of NCC cysts within the patient. Our findings suggest that factors associated with host immune response, such as patient age and sex, as well as possible indicators of previous exposure (presence of calcified cysts at baseline), may play a key role in cyst evolution over time. Identifying factors that are associated with differences in how individual cysts evolve is an important addition to our limited understanding of this parasitic larva. Furthermore, the patterns of differences in cyst evolution by patient and infection characteristics when stratifying on treatment suggest that differences in the host immune response may be impacted by treatment received and future research is needed to assess if these differences indicate different treatment needs. This may lead to improved treatments based on patient characteristics for a more personalized medicine approach, which, hopefully, will improve outcomes. We hope that future research on NCC will include collection and analysis of the evolution of individual NCC cysts rather than looking at cyst evolution aggregated to the patient level to help researchers and clinicians understand what impacts individual NCC cyst evolution and, most importantly, resolution.

## Appendix

**Table:** Summary of models presented in Tables 4–8 and their objectives

Table No.	Model description	Analysis conducted	Research question	Summary of key findings
4	Crude Models	Examined the unadjusted associations of patient (sex and age), infection (2+ cysts and calcified cysts at baseline), and cyst characteristics (location), as well as albendazole treatment, with NCC cyst transitions across phases	What patient, infection and cyst characteristics are associated with cyst evolution without adjusting for other variables?	In the crude models, Infection characteristics (having 2+ cysts at baseline active-degenerating HR = 2.99 [1.28, 9.07] & calcified cysts at baseline degenerating-resolution HR = 2.35 [1.13, 6.67]) as well as Cyst characteristic (parenchymal location degenerating-calcified HR = 1.76 [1.08, 6.50] and calcified to resolution HR = 0.14 [0.01, 0.96] were significantly associated with cyst evolution in the crude models.
5	Multivariable Models	Conducted multiple multivariable models including combinations of covariates (i.e. the variables assessed in model 4) and all adjusted for albendazole treatment to look at independent associations (i.e. adjusted for treatment and covariates)	What patient, infection and cysts characteristics are independently (i.e. adjusted for covariates) associated with cyst evolution.	In full model with all covariates and albendazole, Having calcified cysts at baseline was still significantly associated with evolution from degenerative to resolved (HR = 4.33 [1.24, 367.21]. Having 2+ cysts at baseline and cyst location were no longer significantly associated with cyst evolution
6	Crude Models Stratified by Treatment	Crude models (from Table 4) examined stratified on albendazole treatment.	Does treatment modify the crude association of patient, infection and cyst characteristics on cyst evolution without adjusting for other variables?	In the stratified crude models, The association of cyst evolution with sex differed by albendazole treatment (men had a higher rate transition from degenerative-calcified and degenerative-resolved than women of when treated with albendazole (HR = 2.47 [0.04, 142.56], HR = 2.27 [0.58, 8.48], respectively) but a lower rate when treated with placebo (HR = 0.11 [0.01, 1.60], HR = 0.29 [0.12, 0.60], respectively). Older patients (>40) had a lower rate of transition from active to degenerative (HR = 0.72 [0.18, 2.41]) but higher rate of transition from degenerative to calcified (HR = 1.20 [0.05, 96.24]), degenerative to resolved (HR = 1.87 [0.59, 7.65]) and calcified to resolved(HR = 1.55 [0.57, 7.72]) compared to younger patients when treated with albendazole, but the associations were in the opposite direction among those treated with placebo (HR = 1.53 [0.54, 4.44], HR = 0.12 [0.01, 2.11], HR = 0.31 [0.10, 0.73], HR = 0.63 [0.13, 2.21, respectively). Having multiple cysts at baseline was associated with significantly lower transition rate for the calcified to resolution transition (HR = 0.46 [0.21, 0.94]) but a non-significant higher rate for those in the placebo group (HR = 23.61 [0.52, 84.56]). Among those in the albendazole group, having calcified cysts at baseline remained significantly positively associated with transition from degenerating to resolution (HR = 7.84 [2.81, 858.12]) and slower transition from calcified to resolution (HR = 0.13 [0.01, 0.45]) while among those in the placebo group these transitions were weaker (degenerative to resolve HR = 1.23 [0.47, 4.41) or in the opposite direction (calcified to resolution HR = 21.79 [0.64, 69.24]) but non-significant.
7	Multivariable Models Stratified by Treatment	Stratifying the adjusted models (from Table 5) on albendazole treatment	Does albendazole modify the independent association of patient, infection and cyst characteristics with cyst evolution?	In the stratified full model with all covariates and albendazole, Men had higher rates of transitions across all phases than women when treated with albendazole (active to degenerative HR = 5.78 [0.58, 80.80], degenerative to calcified HR = 10.79 [0.01, 2554.93], degenerative-resolved HR = 2.01 [0.01, 1057.80], calcified to resolved HR = 1.06 [0.01, 226.94]) but lower rates when treated with placebo (HR = 0.29 [01, 2.06], HR = 0.75 [0.01, 86.25], HR = 1.09 [0.10, 3.75], HR = 0.45 [0.01, 13.20], respectively). The pattern with age in the crude model was no longer apparent. Having calcified cysts at baseline was associated with a reduced rate of transition from active to degenerative phase among those treated with albendazole (HR = 0.43 [0.07, 3.82]) but a higher rate among those treated with placebo (HR = 2.11 [0.22, 41.56]).
8	Sensitivity Analysis	Stratifying the adjusted stratified models (from Table 7) but run with the original 4 evolutionary phases collapsed into 2: (1) active to degenerating and (2) degenerating to calcified or resolved.	Does albendazole modify the independent association of patient, infection and cyst characteristics with cyst evolution under the newly constructed outcome?	In the stratified full model with all covariates and albendazole, Men still had higher rates of transition from the degenerative phase than women when treated with albendazole (degenerative to calcified or resolved HR = 1.55 [0.07, 16.47]) but lower when treated with placebo (HR = 0.50 [0.04, 2.28]). Older patients treated with albendazole had higher transition from active to degenerative and lower transition rate from degenerative to calcified or resolved (HR = 0.58 [0.08, 2.29] & HR = 1.46 [0.36, 6.62]) but the opposite direction of association among those in the placebo group (HR = 1.64 [0.38, 5.08] & HR = 0.29 [0.11, 0.72]) Having calcified cysts at baseline was associated with lower transition from active to degenerative among those treated with albendazole (HR = 0.41 [0.05, 3.26]) but higher rate among those treated with placebo (HR = 2.19 [0.17, 40.34]).
